# Notes and News

**Published:** 1898-06-18

**Authors:** 


### NOTES AND NEWS.

(Collected and Communicated.)

Our amusing contemporary, the *Guyoscope*, suggests the following motto for a dental school: "The Tooth, the whole Tooth, and nothing but the Tooth."

In consequence of the resignation of the chaplain of The London Hospital, the Rev. J. D. K. Mahomed, the whole question of the chaplaincy of the hospital is being considered by a special committee.

A highly successful dramatic and musical entertainment, in aid of the funds of the Paulton Cottage Hospital, was given at Midsomer Norton, on Tuesday, 31st ult., by the members of Downside College Choir, Bath, and their musical director, Mr. R. Terry.

The Merchant Taylors' Company have contributed £1,000 to the Prince of Wales's Hospital Fund, and this amount is to be repeated annually during the next two years. The treasurer of St. Mark's Hospital for Fistula, City Road, has received a cheque for £1,000 from Mrs. Douglas Henty to endow a bed to be called the "Julia Henty Bed." The trustees of Smith's (Kensington Estate) Charity have sent a donation of £100 to the funds of the East London Hospital for Children and Dispensary for Women, Shadwell, E.

The new laboratories at University College, Liverpool, which have been erected and equipped by the Rev. S. A. Thompson Yates in the most adequate way for study and research, at a cost of over £25,000, will be formally opened on October 8th. Lord Lister, President of the Royal Society, has consented to perform the opening ceremony, and the Victoria University will take advantage of his visit to Liverpool to confer upon him the honorary degree of Doctor of Science.

The prosecution of a vendor of an instrument professing to cure "deafness and noises in the ear," may prove a warning to an ever-credulous public of the waste of money involved in such quack remedies. The instrument was supplied through the medium of advertisements setting forth in glowing terms, embellished by fictitious testimonials, the marvellous cures wrought by its agency. It was sold at prices varying from 10s. to 15s., and its intrinsic value is said to be 6d.

Dr. Knopf, well known as a scientist and physician, has died at Marienbad. He was 78 years of age.

An anonymous friend has promised £8,000 to build a second block of buildings in connection with the consumption hospitals in Scotland at the Bridge of Weir.

With reference to the announcement last week respecting Mr. E. Mackeson's legacy to the Surgical Aid Society and other societies, the residuary estate amounts to about £110,000, not, as stated in the paragraph, £10,000.

The fourth annual Chemists' Exhibition, organised by the *British and Colonial Druggist*, has during the week been held at the Royal Agricultural Hall, Islington. The exhibition has been well patronised by all sections of the trade, and shows a most satisfactory increase in both the size of the respective stands and in the number of the exhibitors.

A new wing has been added to the Midland Counties Home for Incurables, which was opened recently by the Mayor of Birmingham. The extension scheme will have cost, when complete, £17,000, and provide accommodation for 33 patients. Part of the expense will be defrayed by a Jubilee Commemoration Fund subscribed in Leamington.

The Isolation Hospital for Richmond, Heston, and Isleworth, at Isleworth, has been opened by the Duke of Cambridge. Provision has been made for 46 beds—22 in one ward, and 12, eight, and four respectively in others. At the extreme end of the site a mortuary and post-mortem building is placed, and in the grounds also are stables, laundry, disinfecting building, and ambulance shed. The institution has been erected, at a cost of £13,598, from the design of Mr. Ancell, of 3, Staple Inn.

One of the troubles arising from exalted patronage is that people are apt to take for granted that institutions which are under the wing of Royalty are sure to be well off, whereas many of them are just as hard up as their neighbours. The condition of the National Society for the Employment of Epileptics is a case in point. The rapid development of the colony at Chalfont-St.-Peters, the laying of foundation-stones, and the opening of new houses has no doubt led to the existence of a widespread impression that the society is in a state of extreme prosperity, an impression which is erroneous. The calls upon it are great, and H.R.H. the Duke of York, the president of the society, has desired Mr. Montefiore Micholls, the chairman of the executive committee, to make known how pressing and urgent are the present needs of the institution.

The prospects of the harvest to be gleaned from the Press Bazaar by the London Hospital are excellent. The proprietors of the *Daily Chronicle* have set a splendid example by promising donations of £2,000. A wholesome rivalry is an excellent thing and we naturally wish The Hospital to take a good position in the race. We are hoping for the co-operation of our readers in securing a good supply of fruit wherewith to tempt many purchasers. We expect to have to supplement the kind gifts of fruit promised, and have already received several sums of money for the purpose. But we want still more, and the smallest donation will help to swell the fund and produce, we hope, a large return for the benefit of the London Hospital. Donations addressed to the Manager, 28, Southampton Street, Strand, will be gladly acknowledged.

